# Improving Health Literacy Responsiveness: A Randomized Study on the Uptake of Brochures on Doctor-Patient Communication in Primary Health Care Waiting Rooms

**DOI:** 10.3390/ijerph18095025

**Published:** 2021-05-10

**Authors:** Carel J. M. Jansen, Ruth Koops van ’t Jagt, Sijmen A. Reijneveld, Ellen van Leeuwen, Andrea F. de Winter, John C. J. Hoeks

**Affiliations:** 1 Department of Communication and Information Sciences, Faculty of Arts, University of Groningen, P.O. Box 7600, 9700 AS Groningen, The Netherlands; ruthkoopsvtj@gmail.com (R.K.v.J.); eajvanleeuwen@gmail.com (E.v.L.); j.c.j.hoeks@rug.nl (J.C.J.H.); 2 Language Centre, Stellenbosch University, 44 Banghoek Rd, Stellenbosch 7600, South Africa; 3 Aletta Jacobs School of Public Health, P.O. Box 7600, 9700 AS Groningen, The Netherlands; 4 Department of Health Sciences, University Medical Center Groningen, University of Groningen, P.O. Box 30.001, 9700 RB Groningen, The Netherlands; s.a.reijneveld@umcg.nl (S.A.R.); a.f.de.winter@umcg.nl (A.F.d.W.)

**Keywords:** health literacy responsiveness, organizational health literacy, health literacy, health information, health communication, waiting room, doctor-patient communication, photo story, fotonovela, narrative health communication

## Abstract

Presenting attractive and useful health education materials in waiting rooms can help improve an organization’s health literacy responsiveness. However, it is unclear to what extent patients may be interested in health education materials, such as brochures. We conducted a three-week field study in waiting rooms of three primary care centers in Groningen. Three versions of a brochure on doctor-patient communication were randomly distributed, 2250 in total. One version contained six short photo stories, another version was non-narrative but contained comparable photos, and the third version was a traditional brochure. Each day we counted how many brochures were taken. We also asked patients (*N* = 471) to participate in a brief interview. Patients who consented (*N* = 390) were asked if they had noticed the brochure. If yes (*N* = 135), they were asked why they had or had not browsed the brochure, and why they had or had not taken it. Interview responses were categorized by two authors. Only 2.9% of the brochures were taken; no significant association with brochure version was found. Analysis of the interview data showed that the version with the photo narrative was noticed significantly more often than the non-narrative version or the traditional version. These results suggest that designing attractive and comprehensible health materials is not enough. Healthcare organizations should also create effective strategies to reach their target population.

## 1. Introduction

### 1.1. The Waiting Room as an Opportunity for Improving Health Literacy Responsiveness

The effectiveness of interventions aimed at improving health outcomes depends on multiple factors. These factors not only include an individual’s personal characteristics, but also the individual’s social context, the skills and abilities of the health professionals who are involved, the health system as a whole, and, crucially, also the quality of the communication between the individual and the health professional [1]. By quality of communication we understand the extent to which senders manage to choose the content and form of their messages so that their intentions are well understood by recipients, and the extent to which recipients manage to interpret the messages correctly so that they can respond appropriately [2]. Successful communication between health professionals and patients is possible only if both parties consider it their responsibility to listen carefully and to present the information in a way that it can be clearly understood. Important factors here include avoiding complicated terms or jargon, paying attention, and considering the other person’s perspective.

Adopting this broader perspective on health literacy should result in the development of interventions aimed at levels of influence additional to the level of the individual [3]. Such interventions may improve health literacy responsiveness: “the way in which services, organizations and systems make health information and resources available and accessible to people according to health literacy strengths and limitations.” [4] (p. 6), [5] (p. 433). Improving health literacy responsiveness requires an organization-wide effort to support people in navigating, understanding and using information zin order to take care of their own health [6].

One important setting to improve health literacy responsiveness is the waiting room, the place where the first step in many encounters of patients with health care providers is taken. Health information is often made accessible to patients waiting for their appointment, and social support may occur naturally [7]. An integrative review by Cass and colleagues suggests that providing health-related information in waiting rooms may have an overall positive influence on knowledge, intentions, and behaviors, and may thus be a useful strategy to improve health literacy responsiveness [8].

Patients also appear to value the presence of health information in primary health care waiting rooms. This conclusion is drawn in a questionnaire survey in ten practices in a Belgian town (*N* = 903). Over ninety percent of the patients reported that they often or sometimes read health brochures available in the waiting room, and more than forty percent said they often or sometimes took them home. More than twenty-five percent indicated that leaflets enabled them to ask fewer questions of their doctor and nearly thirty-five percent indicated that leaflets had previously helped them to improve their health-related knowledge and self-management [9].

In a study in the US (*N* = 205) into the influence of the waiting room environment on the perceived quality of care, many comments from patients indicated a preference for waiting rooms that contain “lots to read and look at.” That may help them pass the time and distract them from worrying too much about the health issue for which they are in the waiting room [10]. This conclusion is underscored in another US study (*N* = 320). The outcomes affirm that long waits negatively affect patient satisfaction [11].

There is great variability in the quality, content and amount of health education materials in waiting rooms. A study into the effectiveness of waiting room materials conducted in twenty-seven waiting rooms in a British town (*N* = 556) found substantial variation in the amount, topicality, and quality of health education materials. The authors report that on average, the waiting rooms contained 72 posters covering 23 topics, and 53 leaflets covering 24 topics, with many outdated and poorly presented materials of limited accessibility. 78% of the patients reported that they normally noticed health education materials, while 68% said that they found them useful. Only 47% of the patients agreed that the displays in the waiting rooms were well-designed and attractive. The study also found that the educational level of the patient played an important role in the evaluation of health information products: the lower the educational level, the higher the perceived usefulness and attractiveness of the leaflet or poster [12].

Other studies found that only a minority of patients in waiting rooms read health education materials. In a study in two hospital outpatient waiting areas in Australia (no participants other than counted), the available health information was only infrequently and briefly accessed. The authors suggest that the available health information may not meet the health literacy needs of patients; they also suggest that further research should be conducted to understand how waiting areas may be designed to promote and improve health literacy [7]. In another Australian study, now in a regional general practice (*N* = 74), 16% of the patients reported having read health information about disease prevention in a magazine, while 15% of the patients reported having read a leaflet or poster [13].

All in all, the waiting room can be a valuable opportunity for disseminating health information to improve health literacy responsiveness. One strategy for meeting the needs and abilities of people with different levels of health literacy is to create understandable health education materials [14]. A study from 1998 of the readability of British patient information materials confirms that there is room for improvement here: many leaflets are poorly written [15]. Another British study, conducted in 2015, shows a similar picture. After assessing a total of 345 patient information leaflets, the authors conclude that less than twenty-five percent met recommended reading-level criteria, and that over seventy-five percent were too complex for at least 15% of the English population [16].

However, even if an organization succeeds in providing well-designed health education materials that are easy to understand for a broad audience, these efforts may only lead to changes in knowledge, attitudes and ultimately behaviors if the materials actually reach the members of the target groups for which they are intended. Therefore, it is crucial to find out what characteristics of health education materials, for example offered in a waiting room, can help ensure that they are noticed, read and possibly taken home.

For example, is it conceivable that for patients in a waiting room, “narrative” or “storytelling” versions of health education materials are particularly appealing? Perhaps patients visiting a health care practice are not only motivated to be informed about health-related topics; they may also be looking for something interesting and captivating to read, simply to pass the time or to reduce stress. Below, we briefly discuss why narrative forms of health education in particular can be an engaging and effective way for a broad audience to encounter and then process relevant information. After this, we explain why it is relevant for research to pay more attention to exposure to health information. Then we formulate the aims of this study.

### 1.2. Narrative Health Education Materials, in Particular Photo Stories

Narrative health education materials are increasingly being used to influence health beliefs, and to motivate health behavior change [17,18,19,20,21,22]. Narrative communication uses story structures that provide a mode of communication that people are intimately familiar with. Therefore narrative-based health information could be easier to process [18,23,24]. Health information presented in a narrative format provides both information and entertainment [25,26,27,28]. As stated by Moyer-Gusé and Nabi, for instance, involvement with narrative storylines (“transportation”) and involvement with characters (“identification”) are important determinants of possible persuasive effects of entertainment-education programming [29].

Transportation reduces the ability and the motivation to come up with counterarguments to the persuasive message embedded in the story, because the audience members are immersed in the enjoyable process of being transferred to another world. Identification is also assumed to reduce counterarguing as identification favors adopting the thoughts and feelings of a character instead of criticizing them. Furthermore, identification is expected to increase the extent to which readers feel vulnerable to a given health threat, by contesting the reader’s belief that he or she is uniquely immune to negative consequences, regardless of risky behavior. Furthermore, narratives have the potential to increase personal involvement and provide users with role models and step-by-step scenarios [18]. Narrative communication can thus be viewed as a form of learning through experience [30,31]. Taken together, narrative health communication seems to be a promising strategy for improving the appeal and effectiveness of health literacy interventions.

Recent studies suggest that a specific form of narratives, namely ‘photo stories’ may be particularly effective health communication tools, especially for readers with a low level of literacy [23,32,33,34,35,36]. Photo stories, also called “fotonovelas”, are small publications, often in booklet format, that tell a (dramatic) story by means of photographs and short and easily readable captions. The integrated presentation of textual and visual information in photo stories minimizes cognitive load, thus supporting information processing and learning [37,38]. Studies comparing photo stories to more traditional formats for health communication revealed only small effects on knowledge, attitudes and behavioral intentions, but readers clearly found photo stories more appealing [36,39].

### 1.3. Exposure to Health Information Materials as a Condition for Effectiveness

According to McGuire [40], a series of thirteen steps is necessary for a message to be persuasive. The first step is exposure to the message (“tuning in to the communication”), followed by, among others, the steps “liking”, “comprehension”, “storing knowledge in memory” and “deciding to act”.

Exposure is thus a first and necessary condition for any health education message to be effective. This condition, however, is easily overlooked in communication research, where messages are generally presented to patients in a “forced-exposure” context. In communication studies, attention is often not induced by the materials but by the research context. However, possible effects of processing health messages are only meaningful if members of the target group find such messages appealing enough to actually pick them up and are willing to put effort in reading them. Taking notice of health messages is thus an essential first step in order for these communication products to have any impact on the target group’s behavior.

Research that addresses uptake of health education materials in a natural context is rare and inconclusive. For example, a study in twelve British community pharmacies (where percentages of uptake of health education leaflets ranged between 50% and 72%) found no differences between prominent leaflet display (placed on the desk) and targeted distribution through pharmacy staff [41]. Another British study examined whether patients had read leaflets and posters about oral cancer in an integrated dental unit. Here, 46% of interviewed patients reportedly had read the information; the other patients reportedly had not noticed the posters and leaflets [42]. A small-scale study in a primary health care clinic in a rural town in South Africa compared a photo story on the risks of amphetamine use to a non-narrative traditional brochure on the same subject. Uptake measures over a three week period showed that patients took the fotonovela format more frequently than the traditional brochure [43].

### 1.4. Aims of the Study

As stated above, drawing patients’ attention is an important condition for health communication to be effective. If patients are unwilling to take note of health materials designed to inform and possibly persuade them, the further qualities of these materials become irrelevant.

Therefore, we wanted to study the uptake of health education brochures in a real-life situation in waiting rooms of general practitioners. More specifically, we wanted to determine whether three different versions of a brochure, a short photo story and two alternative versions, would result in differences in how often these different versions would be noticed, browsed, and taken away.

In addition, we wanted to find out why patients did or did not browse a brochure they noticed, why they did or did not take it with them, and how they felt about the different versions of the brochure.

## 2. Materials and Methods

We conducted a field study in three primary health care practices in the Netherlands. During three consecutive weeks, we assessed the uptake of three different versions of a brochure on doctor-patient communication, uptake being defined in terms of numbers of brochures that were noticed, browsed, and taken from the waiting rooms. Furthermore, we interviewed patients during one day of each week about that week’s brochure. A narrative version of the brochure, a non-narrative version containing explicit advice on the same topic, and a traditional version issued by the Dutch Patient Consumers Federation, also containing explicit advice on doctor-patient communication, were distributed across health care practices according to a Latin square design, with daily change of the brochure on display, leading to a balanced random offer of all three brochures; see Table 1.

This procedure ensured that exposure to each of the brochures was equally distributed across the health care practices, so that any uptake differences could be attributed to version of the brochure, rather than for instance, day of the week, or location. The three versions are described in more detail in Section 2.3.

### 2.1. Setting

We performed the study in three primary health care practices in Groningen, a city in the Northern part of the Netherlands. Table 2 shows profiles of these practices.

### 2.2. Patients

During three weeks, we assessed how many brochures were taken away by patients. On the interview days—one day per week for each practice—we approached all patients who were leaving the practice (*N* = 471). 390 of these patients consented to a brief interview. They were first asked if they had noticed the brochure that was at display in the waiting room during their visit. All patients whose response was affirmative (*N* = 135) also answered the follow-up questions they were asked.

### 2.3. Intervention Materials: Three Brochures

We compared a narrative version of a brochure on doctor-patient communication (from here: Photo Stories Version), a non-narrative version containing explicit advice on the same topic (from here: Non-Narrative Version), and a traditional version issued by the Dutch Patient Consumers Federation, also containing explicit advice on doctor-patient communication (from here: Existing Brochure). All three versions of the brochure were designed to help patients address health professionals who lack the skills to properly deal with health literacy issues.

**Figure 1:**
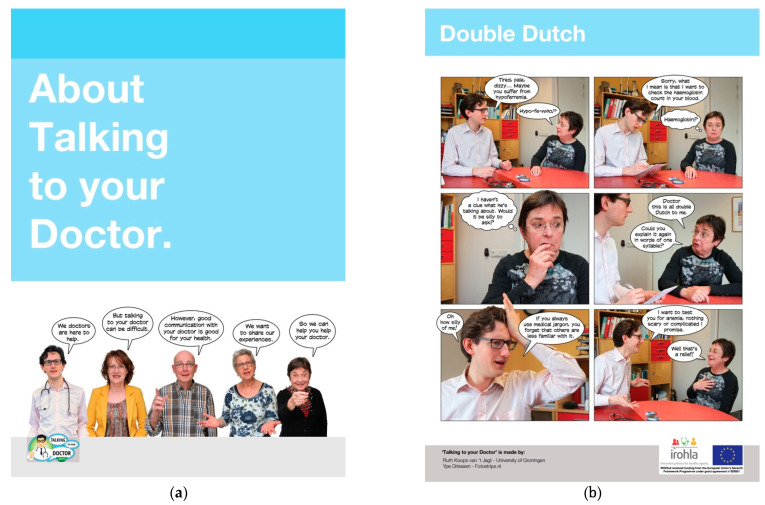
First inside page (panel **a**) and example page (panel **b**) of the Photo Stories Version in English (in this study, the Dutch version was used).

The Photo Story Version contained a set of seven one-page visual narratives. Each page showed a six-frame photo story on a different doctor-patient interaction topic, such as bringing someone to a consultation as support, discussing medication use, and making a question list as preparation for a consultation. The Photo Story Version was developed following a participatory approach which included older adults with limited health literacy [44]. Each topic was incorporated into a brief story using photographs with realistic characters and vivid pictures, and captions and text balloons. See Figure 1 for the first inside page and an example page.

**Figure 2:**
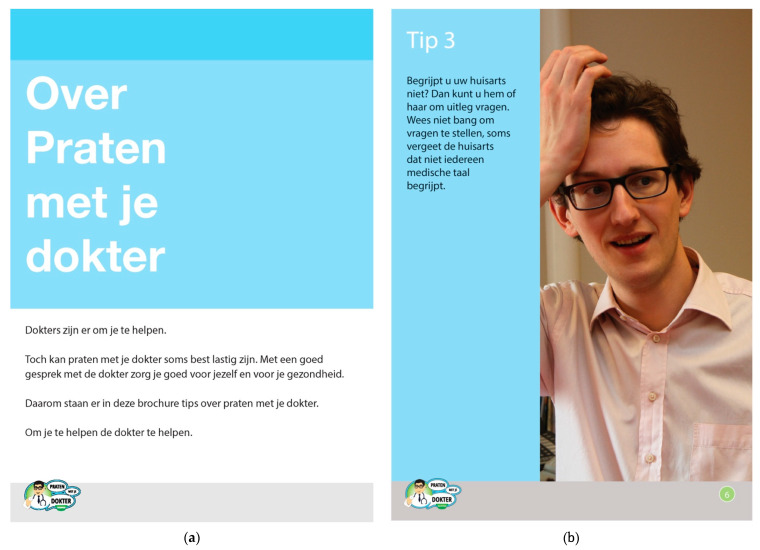
First inside page (panel **a**) and example page (panel **b**) of the Non-Narrative Version (Dutch version; there was no English version). Translation of the title: “About talking to your doctor”. Translation of the text: “Doctors are there to help you. However, sometimes talking to your doctor can be quite difficult. Having a good conversation with your doctor is a way of taking care of yourself and your health. That is why this brochure includes advice about talking to your doctor. To help you help your doctor.” Translation of the text on the example page: “Tip 3”/”Don’t be afraid to ask questions, sometimes the doctor forgets that not everybody understands medical language”.

The Non-Narrative Version contained seven pages on the same topics that were included in the Photo Stories Version. The Non-Narrative Version, however, presented each message that was conveyed as a general advice, without a story line. Each advice was accompanied by one large picture that was selected from the pictures that were used in the Photo Stories Version. The Non-Narrative Version was developed as a “plausible rival” to the Photo Stories Version, using a multiple-feature revision approach [44]. For this purpose, the Photo Stories Version and the Non-Narrative Version were designed using the same colors, paper, size, front page and cover. For the purpose of this study, we presented the Photo Stories Brochure and the Non-Narrative Brochure with the first inside page as front page, because the actual front page of both brochures was identical. See Figure 2 for the first inside page and an example page of the Non-Narrative Brochure.

**Figure 3:**
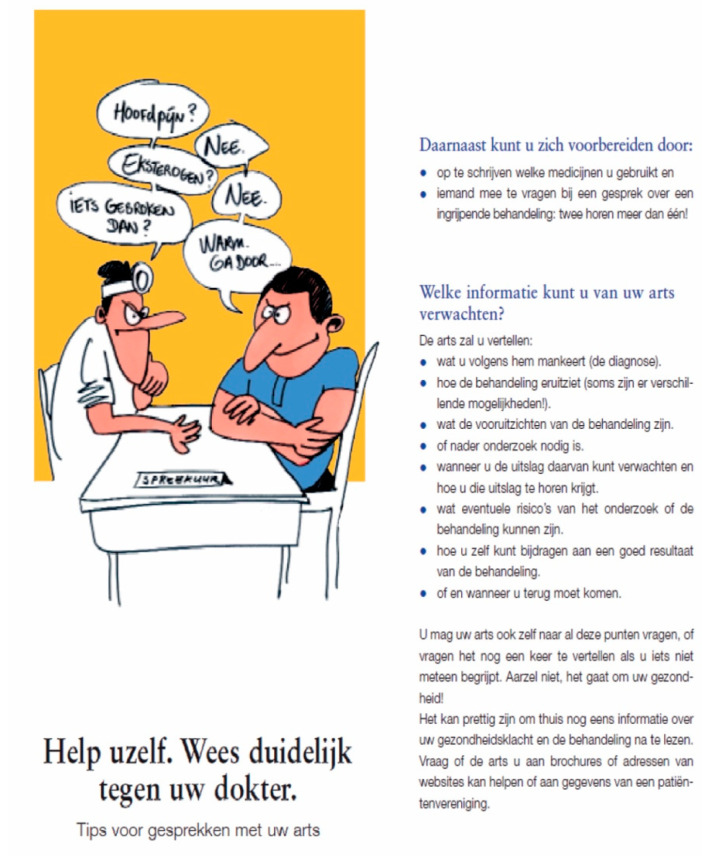
Cover and example page of the Existing Brochure. Translation of the title and the subtitle: “Help yourself. Be clear to your doctor.”/”Tips for conversations with your doctor”. Translation of the first part of the advice on the example page: “In addition, you can prepare yourself by: • writing down what medication you are taking and • asking someone to join you in a conversation about a major treatment: two hear more than one! What information can you expect from your doctor? The doctor will tell you: […]”.

The third version, the Existing Brochure, was issued by the Dutch Patient Consumers Federation. This brochure did not contain any photographs, but it had a cartoon on its cover. The brochure included more text than the other two brochures did. See Figure 3 for the cover and an example page.

To emphasize that patients could take the brochures with them, the front pages sported identical bright yellow stickers mentioning that the brochures could be taken for free.

### 2.4. Procedure and Measurements

Each day, each practice was provided with fixed numbers of one of the three brochure versions, taking into account the size the practice and their waiting rooms (practice A; *N* = 75, practice B; *N* = 45, practice C; *N* = 30). The brochures were placed in display stands (15 brochures per display) on tables or window sills in the practice waiting room. At the end of each day, the numbers of brochures taken away at each of the practices were tallied by research assistants. Unfortunately, because permission for direct observations in the waiting room could not be obtained, we were unable to determine how many leaflets were browsed by patients in the waiting room but not taken.

One day every week, two research assistants plus author EvL approached patients leaving the primary health care practices for a brief interview about the version of the brochure that was on display that day. First the patients were asked if they had noticed the brochure currently on display (Q0). Patients who gave an affirmative answer were then asked to answer some follow-up questions. Consenting patients were consecutively asked Q1: “Did you browse this brochure?”; Q2: “Why/Why not?”; Q3: “Did you take this brochure with you?”; Q4: “Why/Why not?”. In order to collect patients’ opinions on the brochures, they were subsequently asked Q5: “What do you think of this brochure?” and Q6: “Is there anything else you would like to say about this brochure?”.

#### Outcome Measures

The first outcome measure was the *observed number* of brochures that were taken from the primary health care practices per week.

A second set of outcome measures were the *reported numbers* of brochures per day that were noticed, browsed, and taken. Q0 (see above) provided input about the number of brochures that patients said they had noticed, Q1 provided input about the number of brochures they reportedly had browsed, and Q3 provided input about the number of brochures they reportedly had taken with them.

A third set of outcomes concerned the reasons for the patients’ behavior. Participants were asked to explain why they did or did not browse the brochure they had noticed (Q2) and why they did or did not take this brochure with them (Q4). In order to collect participants’ opinions on the version of the brochure they had browsed, they were asked what they thought of the brochure (Q5) and if there was anything else they would like to say about it (Q6).

### 2.5. Analysis

First we assessed differences between the three versions of the brochures in terms of numbers of brochures noticed, browsed and taken, and tested the significance of these differences using Chi-Square tests. After this, we categorized the responses of patients in the interviews. The behavior-related answers to Q2 and Q4 were categorized by two authors: RKv’tJ and EvL as situation-related (e.g., “waiting room too busy”) or self-related (e.g., “too ill to read anything”). The COM-B (Capability, Opportunity and Motivation) model for Behavior change interventions [45] served as the basis for labelling answers to Q2 and Q4 as relevant for motivation (Was the patient motivated to browse the brochure?) or for opportunity (Was the patient able to browse the brochure?). Capability was not regarded to be an issue here. Three key steps from McGuire’s Communication and Persuasion Framework [40] provided the starting point for the categorization of the evaluation-related responses to Q5 and Q6 as relevant for motivation (e.g., “funny”), comprehensibility (e.g., “understandable language”), and possible impact on behavior (e.g., “well prepared after reading”). Any categorization discrepancies were resolved through discussion between RKv’tJ and EvL.

## 3. Results

### 3.1. Background Characteristics of Sample

Of the 471 patients who were approached, 390 (82.8%) consented to a short, structured interview. Out of these 390 patients, 135 (34.6%) reported that they had noticed the brochure: 40 males, 85 females (10 missing answers), mean age 51.21, age range 12–89. Of these 135 patients, 59 were subsequently asked some questions about the Photo Stories Version, 39 about the Non-Narrative Version, and 37 about the Existing Brochure.

### 3.2. Number of Brochures Noticed, Browsed and Taken per Brochure

#### 3.2.1. Observations

Of all available brochures (*N* = 2250), 66 (2.9%) were taken. We found no significant association between brochure version and *observed* number of copies taken from all practices combined: Photo Stories Version: 23, Non-Narrative Version: 20; Existing Brochure: 23 (chi-square(2) = 0.27; *p* = 0.87).

#### 3.2.2. Interviews

A significant relationship was found between brochure version and number of brochures that were *reportedly* noticed (chi-square(2) = 15.30; *p* < 0.001). The Photo Story Brochure was significantly noticed more often than both the Non-Narrative Version (chi-square(1) = 8.39; *p* = 0.003) and the Existing Brochure (chi-square(1) = 13.51; *p* < 0.001). No significant difference was found between the Non-Narrative Version and the Existing Brochure (chi-square(1) = 0.54; *p* = 0.46).

No significant relationships were found between brochure version and number of brochures that were *reportedly* browsed (chi-square(2) = 1.61; *p* = 0.45) or *reportedly* taken (chi-square(2) = 3.14; *p* = 0.21). See Table 3.

### 3.3. Participants’ Responses per Brochure

Table 4 shows the reasons given for browsing or not browsing the brochure. Numbers of reasons given do not equal numbers of patients as patients could give none, one of more reasons.

On the one hand, patients who decided not to browse the brochure on display mentioned that their attention had been elsewhere, such as with their smartphone, or that they were not interested. Nine patients reported that the brochures were out of reach, or that they felt there were too many people in the waiting room. Some patients said they were too tired or too ill to browse the brochure.

On the other hand, patients who did browse the brochure on display reportedly mostly did so because they were interested and curious and regarded the content as relevant, or because they merely wanted to pass the time. In addition, a number of patients mentioned that the brochure on display was appealing and stood out. There were no notable differences among the three versions of the brochure in terms of whether or not they were browsed.

Table 5 shows the reasons given for taking or not taking the brochure. Again, not all patients who were interviewed answered this question; some patients mentioned more than one reason.

Most patients who did not take the brochure that was on display mentioned that they were not interested or that they felt they did not need the information. Another reason reported for leaving the brochure on the display was that reading it in the waiting room had been sufficient. Patients who did take the brochure explained that they were interested in its content, that they took the brochure to read it again or further, or that they took it for someone else. Compared to patients who had gone through the other brochures, relatively more patients who had browsed the Photo Story Version expressed that reading it had been sufficient. Otherwise, there were no notable differences between the three versions in terms of reasons for taking or not taking them.

Patients who reported that they had browsed one of the brochures (see Table 4, second part) were also asked about their opinions on that particular version of the brochure (Q5/Q6). Table 6 shows the opinions expressed about the three versions. Here also, not all patients who were asked this question gave an answer; some patients expressed more than one opinion.

Patients who browsed the brochure expressed mainly positive opinions about all three brochures, related to motivation (“attractive”, “fun”), comprehensibility (“clear”, “understandable”), and effects on knowledge and behavior (“useful”, “great for shy people”). Notably, 21 positive comments were made about the comprehensibility of the Photo Stories Version. Four patients, however, found the Photo Stories Version “childish”, “too simple” or “old-fashioned”. In general, the opinions about the other two versions were less outspoken.

## 4. Discussion

The present study is one of few to explore patients’ responses to various health education materials in a real-life setting. We investigated how patients in a waiting room reacted to three different versions of a brochure on doctor-patient communication. Overall, regardless of brochure version, only few patients noticed, browsed, or took away the brochures. As disappointing as this outcome may be in itself, it is an important finding that it is by no means a given that patients will be interested in the health education materials made available for them.

There was a difference, however, in the salience of the three versions of the brochure tested in this study. According to the responses of the interviewed patients, the version including photo stories was noticed significantly more often than both the non-narrative version and the existing version. Contrary to expectations, however, this version of the brochure was not browsed or taken away significantly more often than the other, non-narrative versions. These outcomes seem to contradict the appeal of narrative forms of health communication often reported in the literature (see, for instance, the reviews in [27,28]. However, reported benefits of narrative health communication are largely based on experimental studies in non-natural settings (see, for instance [22,23,32,33,34,35,36,39]). Although many readers, when asked, say they find a new, narrative form of health education appealing [36,39], this apparently does not automatically imply that they are also willing to process such materials on their own initiative. As suggested by the inconclusive outcomes of earlier studies [41,42,43], situation-related and self-related factors in the specific setting in which the materials are presented influence whether people will undertake the necessary actions to take note of the health messages.

Based on the interviews we conducted, it seems that a number of patients were not interested in the topic of the brochure, or that they were doing something else such as looking at their smartphone. Some patients found the waiting room too crowded, or they said that the brochures were out of reach. Possibly, they felt reluctant to undertake the actions that are necessary to get hold of the brochure (stand up, walk over to and grab the brochure). Enhancing accessibility by removing or minimizing barriers to browse or take away health information materials therefore seems an important factor in improving health literacy responsiveness of waiting room services.

Patients who did browse one of the brochures generally were positive about what they had seen and read. In line with the processing benefits of narrative health information materials reported in previous studies [18,24,25], the version with the photo stories was particularly well received.

### 4.1. Strengths and Limitations

The first and most important strength of our study is that it was not conducted in a in a “forced-exposure” context, but within the natural setting of primary health care practices. Only in such a setting can relevant information be obtained about the attention that patients spontaneously are willing to pay to health education materials: the first necessary condition for whatever possible effect such materials may have.

A second strength of this study is its design. After randomized distribution, patients’ responses to different versions of the same brochures were compared. Furthermore, both self-reports and observations, albeit limited to counting, were used to study patient behavior.

Finally, unlike previous studies about uptake of health education leaflets [38,39,40], the interviews we conducted provided more insight into the reasons why patients were or were not willing to pay attention to health communication materials in a waiting room, and how they felt about the usefulness and attractiveness of these materials.

This study also has some limitations. First, no permission could be obtained for direct observation of the behavior of patients while they were in the waiting room. Although the self-reports obtained in the interviews may give a fair estimate of the differences in the numbers of patients who browsed the brochures, direct observations would have provided more accurate and elaborate information of patients’ browsing behavior. Second, we were not able to collect comprehensive patient characteristics. Therefore, for example, we could not determine whether there were differences between patients with lower and higher levels of health literacy.

Furthermore, presenting the inside pages from the Photo Stories Version and the Non-Narrative Version as the “cover page” (see Figure 1 and Figure 2) may not have been the best choice, as these did not really reflect what the rest of the respective brochures looked like. Perhaps displaying more representative example pages (see also Figure 1 and Figure 2) would have given patients a better idea of what these brochure versions entailed. A further limitation may be that the brochures were printed in A4 format, which may have discouraged people from taking a brochure with them, as it might not easily fit in their pocket or purse.

The categorization of the answers to the interview questions also leaves some room for improvement. In hindsight, more care could have been taken to ensure a clear categorization without possible overlap between the categories.

It is also not entirely clear how representative the group of interview participants was of the population of patients in waiting rooms of general practitioners in the Netherlands. For example, about twice as many women as men were interviewed, and the age of the interviewees was relatively high. It should be noted, however, that in the Netherlands women are on average more likely to see a general practitioner than men. Moreover, most visits to a Dutch general practitioner are made by patients in the age groups of 50 years or older [46].

Furthermore, it is conceivable that the participants sometimes gave socially desirable answers. Although it cannot be ruled out that the results were somewhat influenced by all this, there is no reason to assume that the possible effects differed for the three brochure versions.

### 4.2. Implications for Practice

This study shows that health information materials in a waiting room may easily be overlooked, and that patients who do notice these materials may still want to pass their waiting time with other activities, for instance using their smartphone. Removing any barriers to browse and take health information materials seems crucial. This might, for instance, be achieved by health care professionals actively distributing such materials, or by simply placing multiple displays throughout the waiting room. In addition, other methods can be used to better tailor healthcare delivery to the needs of patients with low levels of health literacy, for instance by strengthening the skills of health care professionals to communicate with these patients [47,48], or by adapting the organization and design of health care services [49].

Furthermore, designers of health education interventions would do well to develop other information media in addition to paper brochures. Given the large number of people who own a smartphone nowadays and who also like to use it when they have to wait somewhere, the obvious option is to make digital health information available on smartphones. This would also prevent COVID 19 related risks that occur when patients start reading a brochure that has been held by someone else shortly before. One way COVID-19 can spread is if someone touches an object that has the virus on it and then touches their own mouth, nose or possibly their eyes.

Conceivably, in the near future, patients entering a waiting room could be automatically alerted to available apps, for example presenting short, preferably interactive, photo stories. The information screens found in many waiting rooms today also offer an interesting option to present health information digitally, for example in the form of short photo stories shown scene by scene, with realistic characters and vivid images.

### 4.3. Implications for Research

Our results show that observations and self-reports may yield complementary results with regard to uptake of health communication materials. Future studies should therefore preferably include data collection methods based on both observations and self-report in order to arrive at a more complete understanding and to overcome disadvantages of using only one measure. Second, we found that noticing brochures does not automatically translate into browsing those materials.

Although not always practically feasible because of reasons of privacy, it seems highly valuable to be on-site and observe patients’ behavior while they spend time in the waiting room.

Most importantly, more research needs to be done in settings where health communication actually takes place. In doing so, researchers may profit from building good relationships with clinic management and staff, from balancing resources with study objectives, and from integrating multiple research methodologies.

## 5. Conclusions

This study shows that providing health information materials in a waiting room may not automatically contribute to the organization’s health literacy responsiveness. Despite positive opinions expressed in interviews, only few patients took the initiative to browse or take away brochures made available in the waiting rooms where this study took place. The narrative version was reportedly noticed more often, and thus performed somewhat better than the other versions did.

Perhaps the best step forward now is to study the spontaneously occurring responses of patients in a waiting room to health information, presented in narrative form or not, via digital media such as smartphones or video screens.

## Figures and Tables

**Table 1:** Study design.

		Primary Health Care Practice A	Primary Health Care Practice B	Primary Health Care Practice C
week 1	Monday	Non-narrative	Photo stories	Existing
	Tuesday	Photo stories	Existing	Non-narrative
	Wednesday	Existing	Non-narrative	Photo stories
	Thursday	Non-narrative	Photo stories	Existing
	Friday	Photo stories	Existing	Non-narrative
week 2	Monday	Existing	Non-narrative	Photo stories
	Tuesday	Non-narrative	Photo stories	Existing
	Wednesday	Photo stories	Existing	Non-narrative
	Thursday	Existing	Non-narrative	Photo stories
	Friday	Non-narrative	Photo stories	Existing
week 3	Monday	Photo stories	Existing	Non-narrative
	Tuesday	Existing	Non-narrative	Photo stories
	Wednesday	Non-narrative	Photo stories	Existing
	Thursday	Photo stories	Existing	Non-narrative
	Friday	Existing	Non-narrative	Photo stories

**Table 2:** Population profiles of participating primary health care practices.

	Primary Health Care Practice A	Primary Health Care Practice B	Primary Health Care Practice C
Number of registered patients	14,157	5284	4465
Male/female (%)	49.2%/50.8%	49.4%/50.6%	48.4%/51.6%
Age range	0–100	0–100	0–99
Number of GPs	8	7	3
Number of other health professionals (nurses etc.)	23	8	6
Average number of GP visits per week	circa 500	circa 350	circa 250
Waiting room characteristics	Large space, where people can enter at two points; many tables with space for leaflets	Medium large space; reading table with space for leaflets	Small space with little room for information leaflets; displays on a small side table and in the window sills.

**Table 3:** Numbers of brochures reportedly noticed, browsed or taken.

	Did you notice this brochure?		
	Yes	No	Total
Photo stories	59 (48.4%)	63 (51.6%)	122
Non-narrative	39 (30.5%)	89 (69.5%)	128
Existing	37 (26.4%)	103 (73.6%)	140
	Did you browse this brochure? (only asked if people had noticed the brochure)		
	Yes	No	Total
Photo stories	18 (30.5%)	41 (69.5%)	59
Non-narrative	14 (35.9%)	25 (64.1%)	39
Existing	16 (43.2%)	21 (56.8%)	37
	Did you take this brochure with you? (only asked if people had noticed the brochure)		
	Yes	No	Total
Photo stories	7 (11.9%)	52 (88.1%)	59
Non-narrative	3 (8.1%)	34 (91.9%)	37 (2 missing values)
Existing	8 (21.6%)	29 (78.4%)	37

**Table 4:** Reasons for browsing or not browsing the brochures, with numbers of respondents giving a specific response (PSV = Photo Stories Version, total *N* = 59; NNV = Non-Narrative Version, total *N* = 39; EB = Existing Brochure, total *N* = 37).

Not browsing the brochure (*N* = 87), because of…			
Situation-related factors		Self-related factors	
Motivation	Opportunity	Motivation	Opportunity
Too many brochures available (PSV:2; NNV:0; EB:0)	Not enough time (PSV:4; NNV:5; EB:2)	Other activities/attention elsewhere, e.g., reading something else, watching children, looking at smartphone, just sitting (PSV:11; NNV:0; EB:7)	Too stressed, distracted, tired or ill (PSV:0; NNV:4; EB:4)
Placing suggested other target group (PSV:1)	Not within reach, too many people in waiting room (PSV:3; NNV:2; EB:4)	Not interested, not relevant, no need, information already known (PSV:13; NNV:12; EB:9)	No reading glasses (PSV:1; NNV:2; EB:1)
		Not a typical reader of brochures (PSV:3; NNV:2; EB:1)	Insufficient Dutch language proficiency (PSV:1; NNV:0; EB:0)
		Only read the front cover (PSV:0; NNV:5; EB:0)	
No particular reason/did not consider/don’t know (PSV:4; NNV:1; EB:1)			
Browsing the brochure (*N* = 48), because of			
Situation-related factors		Self-related factors	
Motivation	Opportunity	Motivation	Opportunity
Within reach (PSV:2; NNV:0; EB:0)		Interested/curious/relevant topic (PSV:9; NNV:5; EB:4)	
Someone else mentioned brochure (PSV:1; NNV:0; EB:0)		Killing time (PSV:4; NNV:1; EB:2)	
Brochure stood out/great appeal compared to other brochures (PSV:1; NNV:3; EB:2)		Great appeal brochure (PSV:3; NNV:1; EB:0)	
Overheard interviewer (PSV:0; NNV:0; EB:1)		Always looking at brochures (PSV:0; NNV:0; EB:1)	
Noticed brochure being put down (PSV:0; NNV:0; EB:1)			

**Table 5:** Reasons for taking or not taking away the brochures with numbers of respondents giving a specific response (PSV = Photo Stories Version, total *N* = 59; NNV = Non-Narrative Version, total *N* = 37; EB = Existing Brochure, total *N* = 37).

Not taking the brochure (*N* = 115), because of…			
Situation-related factors		Self-related factors	
Motivation	Opportunity	Motivation	Opportunity
Not my GP (PSV:1; NNV:0; EB:0)	Not enough time (PSV:2; NNV:0; EB:1)	No interest/need/relevance (PSV:13; NNV:20; EB:15)	Too stressed/distracted/tired/ill: (PSV:1; NNV:0; EB:3)
	Not within reach/waiting room too busy (PSV:2; NNV:0; EB:1)	Reading it was sufficient (PSV:7; NNV:1; EB:0)	Too difficult (PSV:1; NNV:0; EB:0)
	Couldn’t see what it was about (PSV:0; NNV:1; EB:0)	Attention elsewhere (PSV:2; NNV:0; EB:0)	Left it for other people to read (PSV:1; NNV:0; EB:0)
		Not the type of person to take brochures (PSV:1; NNV:0; EB:1)	No reading glasses (PSV:0; NNV:0; EB:1)
No particular reason/did not consider/don’t know (PSV:8; NNV:5; EB:5)			
Taking the brochure (*N* = 18), because of…			
Situation-related factors		Self-related factors	
Motivation	Opportunity	Motivation	Opportunity
		Interested/curious/relevant (PSV:2; NNV:0; EB:1)	To read again further/at (PSV:2; NNV:1; EB:1)
		Took it for someone else (PSV:2; NNV:0; EB:1)	

**Table 6:** Opinions of patients who had browsed the brochures with numbers of respondents giving a specific response (PSV = Photo Stories Version, total *N* = 18; NNV = Non-Narrative Version, total *N* = 14; EB = Existing Brochure, total *N* = 16).

PSV (*N* = 18)	Motivation	Comprehensibility	Possible Impact on Behavior	Other
Positive comments	Not to be missed (1) Fun (4) Attractive (3) Made curious (1) Interesting (1)	Clear, understandable (16) Clear pictures (3) Good font size (1) Accessible (1)	Striking content (1) Beneficial (1) Useful (2) Recognizable situations (5) Thought provoking (2) Important content (2) Great for shy people (1)	Neutral (8) Good brochure (1)
Negative comments	Childish (2) Commercial (1) Old-fashioned (1)	Too simple (1)	Already informed/no need (5)	
NNV (*N* = 14)	Motivation	Comprehensibility	Possible impact on behavior	Other
Positive comments	Interesting (1) Nice (1) Really beautiful (1) Notable, distinct (1)	Clear, understandable (3) Understandable language (3) Clear pictures (1)	Useful (2) Great for people who do not visit doctors regularly (1) Informative (1) Realistic situations (1)	Neutral (3) Good (1) Positive (1)
Negative comments	Not interesting (2) Stinks (1)			
EB (*N* = 16)	Motivation	Comprehensibility	Possible impact on behavior	Other
Positive comments	Distinct/funny cartoon (4) Made curious (1) Colorful (1) Good headline (1)	Good font size (2) Clear, understandable (4) Simple (1) Accessible (2) Good size (1)	Useful (3) Refreshes memory (1) Well prepared after reading (1) Good to draw people’s attention to this topic (2) Good content (1)	Good (1) Neutral (1) Okay (1)
Negative comments	-	-	-	-

## Data Availability

Data (observed and reported number of brochures that are noticed or taken) are contained within the article, see Section 3.2.
